# Endotoxin Translocation Is Increased in Broiler Chickens Fed a *Fusarium* Mycotoxin-Contaminated Diet

**DOI:** 10.3390/toxins16040167

**Published:** 2024-03-25

**Authors:** Nicole Reisinger, Barbara Doupovec, Tibor Czabany, Filip Van Immerseel, Siska Croubels, Gunther Antonissen

**Affiliations:** 1 dsm-firmenich Animal Nutrition and Health R&D Center Tulln, Technopark 1, 3430 Tulln, Austria; barbara.doupovec@dsm-firmenich.com (B.D.); tibor.czabany@dsm-firmenich.com (T.C.); 2 Department of Pathobiology, Pharmacology and Zoological Medicine, Faculty of Veterinary Medicine, Ghent University, Salisburylaan 133, 9820 Merelbeke, Belgium; filip.vanimmerseel@ugent.be (F.V.I.); siska.croubels@ugent.be (S.C.); gunther.antonissen@ugent.be (G.A.); 3 Chair Poultry Health Sciences, Faculty of Veterinary Medicine, Ghent University, Salisburylaan 133, 9820 Merelbeke, Belgium

**Keywords:** broiler, barrier function, deoxynivalenol, endotoxin, fumonisins, gut health, inflammation, tight junction proteins

## Abstract

Broiler chickens in livestock production face numerous challenges that can impact their health and welfare, including mycotoxin contamination and heat stress. In this study, we aimed to investigate the combined effects of two mycotoxins, deoxynivalenol (DON) and fumonisins (FBs), along with short-term heat stress conditions, on broiler gut health and endotoxin translocation. An experiment was conducted to assess the impacts of mycotoxin exposure on broilers, focusing on intestinal endotoxin activity, gene expression related to gut barrier function and inflammation, and the plasma concentration of the endotoxin marker 3-OH C14:0 either at thermoneutral conditions or short-term heat stress conditions. Independently of heat stress, broilers fed DON-contaminated diets exhibited reduced body weight gain during the starter phase (Day 1–12) compared to the control group, while broilers fed FB-contaminated diets experienced decreased body weight gain throughout the entire trial period (Day 1–24). Furthermore, under thermoneutral conditions, broilers fed DON-contaminated diets showed an increase in 3-OH C14:0 concentration in the plasma. Moreover, under heat stress conditions, the expression of genes related to gut barrier function (Claudin 5, Zonulin 1 and 2) and inflammation (Toll-like receptor 4, Interleukin-1 beta, Interleukin-6) was significantly affected by diets contaminated with mycotoxins, depending on the gut segment. This effect was particularly prominent in broilers fed diets contaminated with FBs. Notably, the plasma concentration of 3-OH C14:0 increased in broilers exposed to both DON- and FB-contaminated diets under heat stress conditions. These findings shed light on the intricate interactions between mycotoxins, heat stress, gut health, and endotoxin translocation in broiler chickens, highlighting the importance of understanding these interactions for the development of effective management strategies in livestock production to enhance broiler health and welfare.

## 1. Introduction

Livestock production poses various challenges to broiler chickens, impacting their health and welfare. Stressors such as mycotoxin levels in feed and environmental factors like temperature and humidity have significant implications in this regard.

Mycotoxins are naturally occurring secondary metabolites produced by *Fusarium*, widely found as contaminants in feed and byproducts. Two prominent mycotoxins are deoxynivalenol (DON) and fumonisins (FBs), frequently detected in feed samples. Therefore, the European Union (EU) has established guidance limits for DON and FBs at 5 and 20 mg/kg feed, respectively. However, an increasing number of reports suggest that even levels below these thresholds adversely affect broiler health and performance, and can predispose them to systemic disorders [1,2,3]. Notably, DON not only impacts performance but also affects gut barrier function [4], potentially leading to the translocation of bacteria and bacterial metabolites and molecules into the bloodstream. Consequently, the EFSA panel lowered the reference point for adverse animal health effects for DON from 5 to 0.6 mg/kg feed for broiler chicken and turkey [5]. While information regarding FBs is relatively limited, there are reports demonstrating its negative effects on gut health, including gut integrity and inflammation [4]. Recently, the EFSA panel lowered the reference point for adverse animal health effects for FBs from 20 to 1 mg/kg feed in poultry [6].

In addition to mycotoxins, heat stress has become a growing concern due to climate change and the resulting global warming, leading to more frequent heat stress events. Broilers experience heat stress when subjected to temperatures exceeding their thermoneutral zone, which is depending on their age. Broiler chickens are particularly susceptible to heat stress, due to their elevated metabolic rate and inadequate mechanisms for heat dissipation. Heat stress in broilers encompasses physiological and behavioral alterations, including increased respiration rates, lower feed intake, compromised weight gain, and elevated mortality rates [7]. Furthermore, heat stress affects several aspects of broiler health, including gut barrier function [8,9] and liver health [10,11].

Both mycotoxins and heat stress are known to impair the gut barrier, potentially leading to the translocation of bacterial toxins into the bloodstream. Among these toxins, endotoxins, which are lipopolysaccharides (LPS) present in the outer membrane of Gram-negative bacteria [12], are well known for their detrimental effects on broiler health and welfare once they enter the bloodstream. Endotoxins are naturally present in the gastrointestinal tract, even in healthy animals. However, when the gut barrier is compromised, endotoxin levels in circulation increase, leading to clinical signs such as fever, reduced blood flow, and decreased circulating white blood cells [13]. Although broiler chickens are considered less sensitive to endotoxins compared to other species like pigs or calves [14], several studies have shown that the intravenous or intraperitoneal administration of LPSs triggers an inflammatory response. Furthermore, research has demonstrated that once endotoxins are in circulation, they can impact the gut barrier, establishing a vicious cycle [15,16,17]. Importantly, even in the absence of clinical signs, any activation of the immune system can significantly impact broiler performance and welfare [18].

Limited information is available regarding the effect of mycotoxins on endotoxin concentration in the blood of livestock animals. Studies have primarily focused on DON in pigs, indicating its potential to increase endotoxin concentration in the blood [19] and impair the capacity to detoxify endotoxins [20]. These effects may be attributed to DON’s impact on liver health [21], as the liver is the primary organ responsible for endotoxin detoxification [22]. Currently, no information is available regarding the effects of DON and FBs on endotoxin translocation to the blood in poultry.

Overall, there is a scarcity of studies that investigate the combined effects of different stressors on endotoxin translocation and detoxification. To the best of our knowledge, existing studies have primarily evaluated the effects of mycotoxins or heat stress alone, without considering the combined impact of these factors. Understanding the challenges broiler chickens face during production requires a comprehensive assessment of these intertwined stressors.

The objectives of the present study are twofold: (1) to evaluate the effects of the *Fusarium* mycotoxins DON and FBs on gut inflammation, gut barrier function, and endotoxin translocation; and (2) to investigate whether an additional common stressor like short-term heat stress amplifies the effects of DON and FBs on these parameters.

By examining the influence of mycotoxins and short-term heat stress together, we can gain valuable insights into the complex interactions and potential synergistic effects that may exacerbate the detrimental impact on broiler health and welfare. This research will contribute to a better understanding of the combined challenges faced by broilers in the context of mycotoxin exposure and environmental stressors, ultimately informing strategies for improved management and mitigation in livestock production.

## 2. Results

### 2.1. Growth Performance

Broilers were divided into three groups: a control group receiving a control diet, a group receiving a diet contaminated with DON (3070 µg DON/kg), and a group receiving a diet contaminated with FBs (12,900 µg FB_1_ + FB_2_/kg). There was no difference in the body weight on day 1 (*p* > 0.05; Table 1). The results showed a decrease in body weight gain in broilers fed the DON-contaminated diet from day 1 to 12 (13%), compared to the control group (*p* < 0.05). In addition, there was a decrease in body weight gain in broilers fed the FB-contaminated diet over the whole trial period from day 1 to 24 (9%) compared to the control group (*p* < 0.05) (Table 1).

### 2.2. Thermoneutral Conditions—Endotoxin Activity of Intestinal Digesta

**Figure 1:**
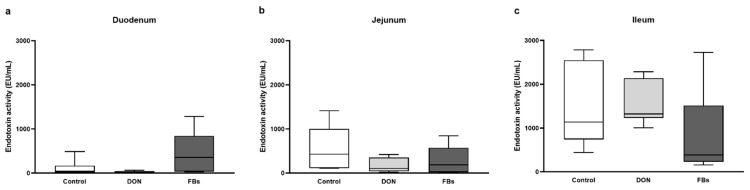
Intestinal endotoxin activity [EU/mL] of the (**a**) duodenum, (**b**) jejunum, and (**c**) ileum of broiler chickens fed a control diet, a deoxynivalenol (DON)-contaminated diet, or a fumonisin (FB)-contaminated diet for 25 days at thermoneutral conditions. *n* = 6 animals/treatment.

At thermoneutral conditions, the endotoxin activity in the duodenum, jejunum, and ileum was not affected by the DON- or FB-contaminated diet (*p* > 0.05; Figure 1).

### 2.3. Thermoneutral Conditions—Gene Expression of Intestinal Tissue

On day 25, gene expression analyses were conducted on the mid-duodenum, mid-jejunum, and mid-ileum tissues for selected genes related to gut barrier function and inflammation, including Toll-like receptors (TLR2 and TLR4). In the duodenum, there was only a trend towards an increase in IL6 expression in broilers fed the DON-contaminated diet (*p* = 0.0519; Table 2). No effects of the DON-contaminated diet were observed on the expression of the selected genes in the jejunum (*p* > 0.05; Table 2). However, in the ileum, there was a trend for an increase in the expression of ZO2 in the animals fed the DON diet compared to the control group (*p* = 0.0803; Table 2). There were no effects of the FB-contaminated diet on the expression of the selected genes in the duodenum (*p* > 0.05; Table 2) and ileum (*p* > 0.05; Table 2). In the jejunum, a decrease in the expression of tight junction proteins (CLDN5, OCLN, and ZO2) as well as a decrease in TLR4 expression were observed in the FB-contaminated group compared to the control group (*p* < 0.05; Table 2).

### 2.4. Thermoneutral Conditions—Endotoxin Marker (3-OH C14:0)

**Figure 2:**
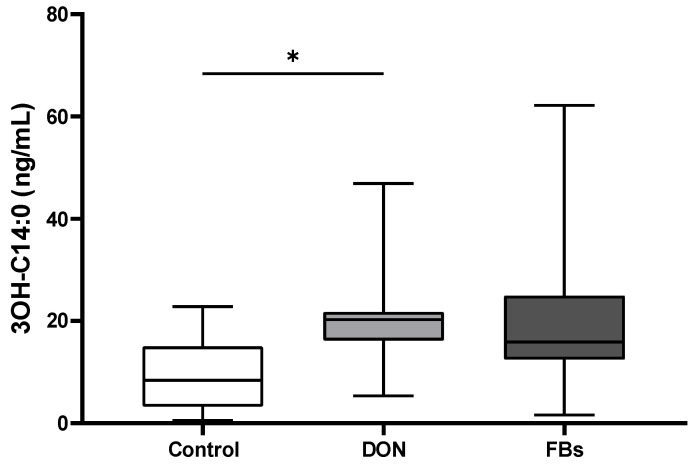
Mean plasma 3-OH C14:0 concentration (ng/mL) of broilers fed a control diet, a deoxynivalenol (DON)-contaminated diet or a fumonisin (FB)-contaminated diet for 25 days. Animals were kept under thermoneutral conditions. *n* = 12 animals/treatment. Values are presented as mean with standard deviation. * Superscripts indicate significant difference (*p* < 0.05).

The plasma concentration of the endotoxin marker (3-OH C14:0), which is a hydromyristic fatty acid specific for endotoxins, was measured using LC-HRMS. The results showed an increase (2.1-fold) in plasma 3-OH C14:0 concentration in broilers fed the DON-contaminated diet (*p* < 0.05; Figure 2).

### 2.5. Heat Stress Conditions—Endotoxin Activity of Intestinal Digesta

**Figure 3:**
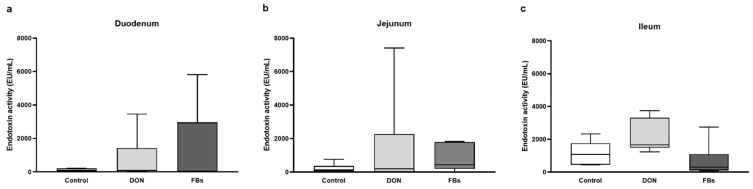
Intestinal endotoxin activity [EU/mL] of the (**a**) duodenum, (**b**) jejunum, and (**c**) ileum of broilers fed a control diet, a deoxynivalenol (DON)-contaminated diet, or a fumonisin (FB)-contaminated diet for 25 days. Animals were kept under heat stress conditions (34 °C) on day 26 for 10 h. *n* = 6 animals/treatment. Values are presented as mean with standard deviation.

When broilers were exposed to heat stress for 10 h and fed a diet contaminated with DON or FBs, there was no effect on the endotoxin activity in the duodenum, jejunum, or ileum (*p* > 0.05; Figure 3).

### 2.6. Heat Stress Conditions—Gene Expression of Intestinal Tissue

On day 26, all animals were exposed to heat stress for 10 h. Following heat stress, gene expression analyses were conducted on the mid-duodenum, mid-jejunum, and mid-ileum to evaluate selected genes related to gut barrier function and inflammation, including Toll-like receptors.

Under heat stress conditions, there was no effect of the DON-contaminated diet on the expression of any gene in the duodenum or ileum compared to the control group (*p* > 0.05; Table 3). In the jejunum, the DON-contaminated diet led to a decrease in IL8 expression (*p* < 0.05; Table 3).

For the FB-contaminated diet under heat stress conditions, in the duodenum, the expression of CLDN1, TLR2, and IL6 was increased compared to the control group (*p* < 0.05; Table 3). In the jejunum, the FB-contaminated diet resulted in a decreased expression of CLDN5 and ZO1 (*p* < 0.05), and there was a trend towards a decreased expression of OCLN (*p* = 0.0528; Table 3). Additionally, the expression of TLR4 and IL1b in the jejunum was decreased (*p* < 0.05; Table 3), and there was a trend towards a decreased expression of TLR2 (*p* = 0.0802; Table 3). In the ileum, the expression of CLDN5 and ZO2 tended to be increased (*p* < 0.06, Table 3). In addition, the expression of TLR4 was increased for broilers fed the FB-contaminated diet (*p* < 0.05; Table 3).

### 2.7. Heat Stress Conditions—3-OH C14:0 Plasma Concentration

**Figure 4:**
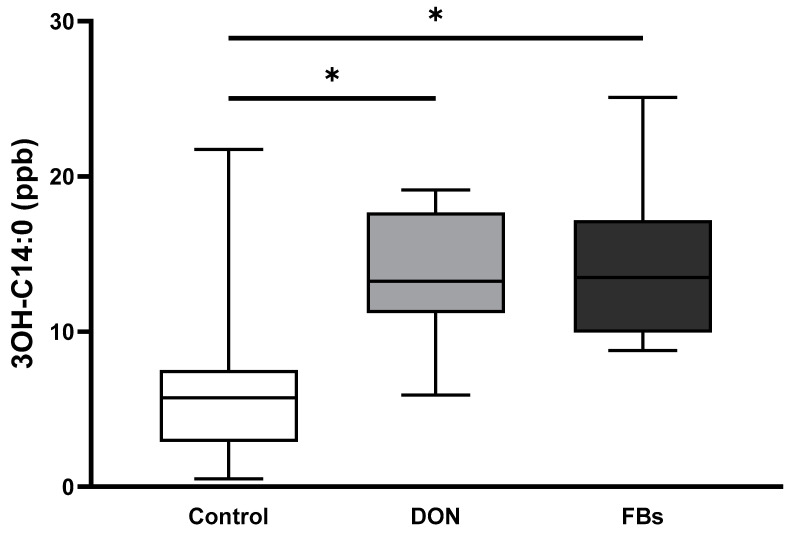
Mean plasma 3-OH C14:0 concentration (ng/mL) of broilers fed a control diet, a deoxynivalenol (DON)-contaminated diet, or a fumonisin (FB)-contaminated diet for 25 days. Animals were kept under heat stress conditions (34 °C) on day 26 for 10 h. *n* = 12 animals/treatment. Values are presented as mean with standard deviation. * Superscripts indicate significant difference (*p* < 0.05).

In animals exposed to heat stress, the results of the LPS marker 3-OH C14:0 showed that broilers fed the DON-contaminated diet exhibited a 2.1-fold increase in plasma 3-OH C14:0 concentration compared to the control group (*p* < 0.05; Figure 4). Similarly, broilers fed the FB-contaminated diet showed a 2.3-fold increase in plasma 3-OH C14:0 concentration compared to the control group (*p* < 0.05; Figure 4).

## 3. Discussion

Broilers are constantly exposed to various challenges throughout their lifecycle in livestock production, significantly impacting their health and welfare. Therefore, it is crucial to evaluate the combined effects of different stressors to replicate the conditions and challenges these animals face on commercial farms. In our study, we aimed to assess, for the first time, the effects of mycotoxins combined with heat stress not only on gut health but also on the translocation of endotoxin, which can have severe detrimental effects by triggering an inflammatory response.

Initially, we examined the effect of two main mycotoxins, DON and FBs, on the body weight of broilers. As expected, DON (3 mg/kg feed) led to a reduction in body weight gain; however only in the starter phase (Day 1–12). Intriguingly, FBs (13 mg/kg feed) also resulted in decreased body weight gain, over the whole trial period (Day 1–24). These findings underscore the fact that mycotoxins can exert significant effects on broilers health and corroborates the updated opinion of the EFSA panel to lower the reference levels from 5 to 0.6 mg/kg feed DON for broiler chicken and turkey, and from 20 to 1 mg/kg FBs for poultry [5,6].

Due to the trial design, we were unable to directly assess feed intake, which could have provided insights into whether the performance losses were solely attributed to reduced feed intake due to mycotoxins or if energy consumption was diverted towards supporting the immune system. Glucose is one of the primary fuels for the immune system, and studies in dairy cows and pigs have demonstrated increased glucose demand following the intravenous application of endotoxins. For instance, in dairy cows, it was estimated that over 1 kg of glucose was required within 720 min [23], and in pigs, approximately 116 g of glucose was needed within 480 min [24] using the euglycemic clamp method. Although similar data are not available in broilers, evidence from cattle and pigs strongly suggests that the presence of endotoxins in the bloodstream leads to a nutrient repartitioning away from growth in favor of supporting the immune system.

Besides the well-known effects of DON and FBs on performance, their impact on the gut microbiome is less well understood. However, recent studies have demonstrated that DON can alter the composition of the jejunal [25] and cecal [26] microbiota, as assessed through 16S rRNA gene sequencing. Additionally, Antonissen et al. [27] showed that FBs can influence the intestinal microbiota in the ileum. The microbiome is of particular interest as changes in its composition can lead to the release of endotoxins, either due to bacterial lysis or the excessive growth of Gram-negative bacteria. Therefore, we aimed to investigate the levels of endotoxins present in the gut under both non-challenged and challenged conditions. To assess this, we measured the endotoxin activity in the duodenum, jejunum, and ileum using the *Limulus Amebocyte* Lysate (LAL) assay, as commonly done in other species, such as ruminants [28,29,30].

Overall, our results confirmed previous findings [31,32] that endotoxin activity in the digesta increases from the duodenum to the ileum in untreated animals. Interestingly, we did not observe any significant effects of DON and FBs on endotoxin activity in the digesta of any intestinal segment. However, this may be attributed to the low sample size and the potential inter-individual variability between animals. Additionally, it is plausible that heat stress itself played a role in influencing endotoxin activity. Heat stress has been shown to exert a considerable influence on the gut microbiome, potentially affecting the release of endotoxins from Gram-negative bacteria. Liu et al. [33] demonstrated a significant impact of heat stress on the jejunal microbiome in yellow-feathered broilers, which may be associated with dysbiosis. Furthermore, other studies have confirmed the influence of heat stress on the cecal microbiome [34].

It is important to note that the LAL assay, which we utilized in this analysis, cannot differentiate between endotoxins originating from pathogenic or commensal bacteria. This distinction is crucial because the structure of the lipid A component, which is the reactive part of endotoxins, can vary (e.g., in terms of the number of phosphate groups or 3-OH C14 groups), leading to variations in immune response activation [12]. To gain further insights into the structure of endotoxin molecules present in the digesta of animals subjected to different treatments (such as mycotoxins and heat stress in our study), more advanced techniques like matrix-assisted laser desorption ionization time-of-flight mass spectrometry (MALDI-TOF MS) would be required. While MALDI-TOF MS has been discussed in relation to ruminants [35], its application in other species has yet to be explored.

For future studies, it would be interesting to include microbiome data from the duodenum, jejunum, and ileum to investigate correlations with endotoxin activity in the digesta. Furthermore, a more comprehensive characterization of the types of endotoxins present based on structural analytics, such as MALDI-TOF MS, could provide valuable insights.

The gut barrier plays a crucial role in preventing the translocation of endotoxins from the intestine into the bloodstream, even in healthy animals where endotoxins are naturally present at certain concentrations. In our study, we observed no significant effect of DON on the gene expression of tight junction proteins, which are important for maintaining gut barrier integrity. Intriguingly, FBs had a more pronounced impact on genes related to gut barrier function and inflammation. Particularly during heat stress, FBs exhibited a strong influence on genes associated with gut barrier function, although the effect varied depending on the specific region of the intestine. In the jejunum, FBs downregulated the expression of tight junction proteins like CLD5 and ZO2 under heat stress conditions, while in the ileum, FBs upregulated the expression of these genes. These findings highlight the complex regulatory mechanisms underlying gene expression related to the gut barrier.

Overall, the literature provides conflicting results regarding the effects of DON and FBs on gut barrier function. Some studies have reported both the upregulation and downregulation of genes associated with gut barrier function in response to mycotoxin exposure [36,37,38]. For instance, a recent study by Paraskeuas et al. [4] found only minor effects of feeding DON and FBs to broilers for 39 days on genes related to gut barrier function in the ileum, while the duodenum and jejunum were unaffected. However, it is important to consider that gene regulation is a dynamic process, and the timing of sample collection may play a crucial role in explaining differences in the outcomes of different studies.

Mycotoxins have also been described to affect genes associated with intestinal inflammation [39]. Therefore, we evaluated the effects of DON and FBs on the expression of genes related to the inflammatory response in the intestine in our study.

Overall, our study revealed that mycotoxins had the strongest effect on the inflammatory response in the jejunum, followed by the ileum and duodenum. However, under thermoneutral conditions, only the expression of TLR4 was downregulated in the jejunum by FBs, while no significant effects were observed for DON and FBs on the expression of other inflammatory genes. The downregulation of TLR4 in the jejunum could be attributed to a negative feedback loop; for instance, elevated levels of IL10 may be capable of downregulating TLR4 expression [40]. Additionally, microRNA 181a has been suggested to provide a negative feedback regulation of endotoxin-induced inflammation in macrophages [41], which are important resident immune cells in the intestine. Another possible explanation is the suppression of the immune response due to FBs [42]. A study by Bouhet et al. [43] described a decrease of IL1b in vivo and in vitro due to FBs [44].

This immune suppressive effect by FBs was also evidenced in our study by the downregulation of IL1b and IL8 in animals receiving FBs under thermoneutral conditions, along with a downregulation of IL1b under heat stress conditions in the jejunum. In contrast, the ileum exhibited a different pattern, with an increased expression of TLR4 and genes related to gut barrier function. This differential response of genes involved in gut barrier function and inflammation could be attributed to variations in blood supply to different parts of the gut during heat stress, as well as the higher sensitivity of the upper parts of the gut to mycotoxin-contaminated feed.

Another interesting finding was the increased expression of TLR2 in the duodenum in broilers exposed to heat stress conditions and receiving the FB-contaminated diet. TLR2 is known to be involved in the inflammatory pathway and immune response. This upregulation of TLR2 in the duodenum may indicate an increased inflammatory response, which could be contributing to the significant upregulation of IL6 (by 3.7 fold) observed in these animals.

Furthermore, DON was found to downregulate the expression of IL8 in the jejunum under heat stress conditions, indicating its potential immunosuppressive effect, which has been previously described for pigs fed a low dose of DON [44].

Our findings highlight the complex and tissue-specific effects of mycotoxins on the inflammatory response in the intestine, suggesting the need for further investigation into the underlying mechanisms and potential interactions between mycotoxins, heat stress, and gut physiology.

In our study, we also assessed the levels of 3-OH C14:0 in the blood, which is a specific fatty acid, present in the lipid A part of endotoxins and can be used as a direct chemical marker for endotoxins. This approach offers an advantage over conventional methods like the LAL assay for detecting endotoxins in plasma, as it is structure-based and less sensitive to interferences from other plasma proteins. Furthermore, it enables the direct detection of the total amount of endotoxins, including both free and bound forms [45].

Under thermoneutral conditions, only broilers fed with DON showed a two-fold increase in the plasma levels of 3-OH C14:0. However, during heat stress conditions, both DON and FBs resulted in a two-fold increase in 3-OH C14:0 levels. Interestingly, another study also demonstrated that the combination of heat stress with oral application of endotoxins led to a significant increase in 3-OH C14:0 levels in the blood [32]. This highlights the potential of 3-OH C14:0 as a marker for endotoxin translocation to the blood. Nevertheless, as these are currently the only two studies utilizing 3-OH C14:0 as a marker for endotoxin concentration, further research is necessary to validate these findings. Additionally, measuring other markers related to systemic inflammation, such as cytokines and eicosanoids, may provide valuable insights.

In addition to gut barrier function, liver health is crucial for endotoxin detoxification. Several studies have described the negative impact of heat stress on liver health in poultry [11,46,47]. However, to the best of our knowledge, there is currently no study available evaluating the effects of liver health and mycotoxins on endotoxin clearance in broilers. Nonetheless, a study by Kahlert et al. [20] administered LPS intravenously to pigs fed with DON, which resulted in higher levels of endotoxins in the blood and a delay in endotoxin clearance. Furthermore, FBs have been described as hepatotoxic. Therefore, we speculate that liver health may have contributed to the higher 3-OH C14:0 levels observed in broilers fed mycotoxins, particularly during heat stress.

In summary, our study illustrated that the presence of DON and FBs, whether independently or in combination with short-term heat stress conditions, had a substantial impact on broiler health. However, given the common co-contamination of DON and FBs, it is even more important to evaluate the combined effects of both mycotoxins on animal health under heat stress conditions. However, as our trial design did not allow for direct comparison between thermoneutral conditions and heat stress conditions in combination with mycotoxin-contaminated diets, future research should aim to further investigate the interactions between mycotoxins, heat stress, gut barrier function, liver health, and systemic inflammation to better understand the mechanisms underlying their combined effects and their impact on animal health.

## 4. Conclusions

In conclusion, our study provides evidence that feeding broilers a DON- or FB-contaminated diet has a significant impact on growth performance and the expression of genes associated with gut barrier function and inflammation. Moreover, mycotoxins were found to increase the translocation of endotoxins in chickens, which poses additional risks to broiler health. Importantly, heat stress exacerbates the effects of mycotoxins on endotoxin translocation, underscoring the need to consider the combined influence of the exposome, including environmental factors such as mycotoxins and temperature.

These findings highlight the importance of implementing effective mitigation strategies to reduce mycotoxin contamination and manage heat stress in broiler production. By addressing these factors, we can minimize the negative impact on growth performance, gut health, and overall broiler welfare. Future research should focus on developing targeted interventions to mitigate the detrimental effects of mycotoxins and heat stress, ultimately improving the health and productivity of broiler chickens.

## 5. Materials and Methods

### 5.1. Ethic Statement

The animal experiments were approved by the ethical committee of the Faculty of Veterinary Medicine and the Faculty of Bioscience Engineering of Ghent University (EC 2019/68, approval date: 17 October 2019).

### 5.2. Animals and Trial Set-Up

The experiment was performed with a total of 252 one-day-old Ross 308 male broilers, which were obtained from a commercial hatchery (Vervaeke-Belavi, Tielt, Belgium). Animals were placed randomly into 18 pens (14 birds/pen). Pens (2 m^2^) contained a clean wood shavings litter (2.5 kg/m^2^), one drinker line with 5 nipples in total, and two feeder pans. Broilers had ad libitum access to feed and water. Up to day 3, the broilers were subjected to a light schedule of 23 h light and 1 h darkness. From day 4 onwards, the animals were subjected to a light schedule of 18 h light and 6 h darkness.

Animals were fed a commercial wheat-based pre-starter crumble feed for the first four days. Thereafter, a commercial mash control or mycotoxin-contaminated wheat-based grower diet was fed until the end of the trial. Animals were fed according to the Ross 308 recommendations.

Pens were divided into three diet groups: Control, DON (3 mg/kg feed), and FB (13 mg/kg feed) group. Mycotoxin-contaminated feed was produced by adding DON or FB culture materials to a control diet. These DON and FB culture materials were commercially available purified and crystallized culture material of *Fusarium graminearum* and *Fusarium verticillioides*, respectively (Biopure–Romer Laboratories Diagnostic GmbH, Tulln, Austria). The mycotoxin contamination of control and contaminated diets was assessed by an accredited commercial laboratory (Primoris, Zwijnaarde, Belgium) using a validated multi-mycotoxin liquid chromatography–tandem mass spectrometry method (LC-MS/MS). Samples were taken at three different locations in each batch, subsequently pooled per batch, and analyzed for mycotoxin contamination. The samples were tested for the following mycotoxins: aflatoxin B1, aflatoxin B2, aflatoxin G1, aflatoxin G2, cytochalasin E, DON, 3 + 15 acetyl-deoxynivalenol (3 + 15 ADON), sum of T-2 and HT-2 toxins, nivalenol, fumonisins B1 + B2 (FB1 + FB2), and zearalenone (ZEN). Trace amounts of DON and FB1 + FB2 were detected in the pre-starter feed (25 µg DON/kg and 44 µg FB1 + FB2/kg feed, respectively). Traces of DON (41 µg/kg feed) and FB1 + FB2 (47 µg/kg feed) were detected in the control grower diet. In the DON-contaminated grower diet, 3070 µg DON/kg feed was detected; trace amounts of 3 + 15 ADON (230 µg/kg), ZEN (48 µg/kg), and FB1 + FB2 (133 µg/kg) were also observed. In the FB-contaminated grower diet, 12,900 µg FB1 + FB2/kg feed was detected, and also trace amounts of DON (28 µg/kg) and ZEN (46 µg/kg).

Body weight was recorded on days 1, 12, and 24.

**Figure 5:**
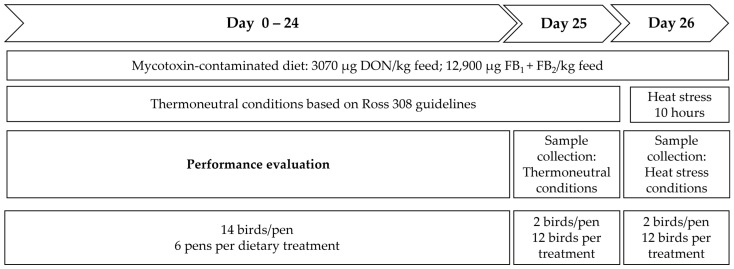
Experimental design: a total of 252 birds (14 birds/pen, 6 pens/dietary treatment) were fed the experimental diets (Control, DON-contaminated diet, or FB-contaminated diet) throughout the whole trial period (Day 0–26). On day 25, 2 birds per pen (12 birds/treatment) were sampled under thermoneutral conditions. Thereafter, heat stress was applied for 10 h, and at the end, blood samples from 2 birds per pen (12 birds/treatment) were collected.

Temperature was kept over 25 days at thermoneutral conditions, with a room temperature of 33 °C during the first week of the experiment, and reduced thereafter until 24 °C was reached on day 25. On day 26, all birds were kept under short heat stress conditions, as an additional stressor. Therefore, temperature was gradually increased from 24 °C to 34 ± 2 °C within one hour, and temperature was subsequently maintained at 34 ± 2 °C for a period of 10 h, with a relative humidity (RH) of 55–65%. The trial design is outlined in Figure 5.

### 5.3. Sampling

On day 25 (thermoneutral conditions) and day 26 after 10 h of heat stress (heat stress conditions), EDTA blood was withdrawn from the jugular vein from two animals per pen. Immediately following blood sampling, these animals were euthanized by an intravenous injection of an overdose of sodium pentobarbital (Natrium Pentobarbital 20%, Kela Veterinaria, Sint-Niklaas, Belgium). The plasma was separated and stored at −20 °C in ENDOGRADE^®^ tubes (Biomerieux Austria GmbH, Vienna, Austria). Digesta and tissue were sampled from the mid-duodenum, mid-jejunum, and mid-ileum from one animal per pen. Digesta (~1 g) was frozen in endotoxin-free Eppendorf tubes (Eppendorf, Hamburg, Germany). Tissue (~50 mg) was stored in RNA later overnight before storage at −80 °C.

### 5.4. Analysis

#### 5.4.1. Endotoxin Analysis Gastrointestinal Tract

Endotoxin analysis of digesta from the duodenum, jejunum, and ileum was performed as previously described [32]. Briefly, digesta samples were homogenized, diluted, and centrifuged before the analysis of the supernatant with the Chromogenic limulus amebocyte lysate (LAL) assay (Charles River, Wilmington, MA, USA).

#### 5.4.2. RNA Extraction and Gene Expression Analysis of the Intestinal Tissue

RNA extraction from small intestinal segments was performed as previously described [38]. Briefly, total RNA was isolated using the RNeasy Mini Kit (Qiagen Ltd., Crawley, UK), according to the manufacturer’s instructions. RNA was treated with the Turbo DNA-free kit (Ambian, Austin, TX, USA), as per the manufacturer’s instructions, to remove genomic DNA contamination. The RNA concentration was measured by absorbance at 260 nm with a Nanodrop^®^ spectrophotometer (Thermo Scientific, Wilmington, MA, USA). Reverse transcription was carried out using the iScript cDNA Synthesis Kit (Bio-Rad Laboratories, Nazareth Eke, Belgium). The obtained cDNA was stored at −20 °C until further analysis.

The primer sequences used for CLDN1, CLDN5, OCLN, ZO1, ZO2, TLR2, TLR4, IL1β, IL6, IL8, ACTB, GAPDH, G6PD, and RPL7 (Table 4) were obtained from the published literature [3,38,48]. The specificity of each primer set was checked using Nucleotide BLAST (Nucleotide Basic Local Alignment Search Tool; https://blast.ncbi.nlm.nih.gov/Blast.cgi; accessed on 1 May 2020) and by performing a standard PCR (40 cycles) on a pooled cDNA mix (diluted 1:5). The pooled cDNA mix comprised 2 μL of cDNA from 18 different samples per segment, with 6 per experimental group, giving a total volume of 36 µL. The PCR products were checked using 1.5% (*w*/*v*) agarose gel electrophoresis, and single band amplification was confirmed. Primer efficiency was evaluated using serial dilutions of the pooled cDNA mix (1:5, 1:25, 1:125, and 1:625). For every standard curve, we assessed the amplicon efficiency (E), correlation coefficients (R^2^), and the slope. Water and no-template samples were used as negative controls for each primer set. The melting curves were also analyzed, and for all primer pairs, a single peak was detected. Reference genes were evaluated for all experimental groups and all different tissues. The three most stable reference genes, glyceraldehyde-3-phosphate dehydrogenase (GAPDH), glucose-6-phosphate dehydrogenase (G6PDH), and Ribosomal protein L7 (RPL7), were selected using geNorm software. RT-qPCR was performed using SYBR-Green Supermix (Bio-Rad Laboraties) in a Bio-Rad CFX-384 system. Each reaction was carried out in triplicate in a 12 mL total reaction mixture using 2 mL of the cDNA sample and 0.5 mM final qPCR primer concentration. The q-PCR conditions used were 1 cycle of 95 °C for 10 min, followed by 40 cycles of 95 °C for 20 s, 58 °C for 30 s, and a stepwise increase of the temperature from 65° to 95 °C (at 0.5 °C/5 s). The results were analyzed using the Bio-Rad CFX manager 3.1. Quantification cycle (Cq) values were obtained using auto baseline settings, and these were applied per primer set. These raw Cq values were imported in QBase 9 [49] for fully automated analysis. The results are shown as fold changes of mRNA expression, which were calculated based on the calibrated normalized relative quantity (CNRQ) values obtained in QBase.

#### 5.4.3. Plasma Endotoxin Marker (3-OH C14:0)

Endotoxin concentration was measured based on the 3-OH C14:0 concentration in plasma. A modified version of the method described by Pais de Barros et al. [44] was used. Briefly, each sample was prepared twice to measure unesterified and esterified 3-OH C14:0. To both samples, 5 µL of 3-OH C14:0-d5 (Eurisotop, Saint-Aubin, France) dissolved in absolute ethanol (Chem-Lab NV, Zedelgem, Belgium; 1 µg/mL) was added to calculate the recovery of 3-OH C14:0. To measure unesterified 3-OH C14:0, 100 µL plasma was mixed with 300 µL of endotoxin-free water before extraction. To measure esterified 3-OH C14:0, 100 µL plasma was hydrolyzed with 300 µL of 8 M HCl for 1 h at 90 °C. Thereafter, 100 µL of 10 M sodium hydroxide was added to all samples before 3-OH C14:0 was extracted with ethyl acetate. Thereafter, extracts were dried with a SpeedVac (Eppendorf) and dissolved in 100 µL of ethanol.

The extracts were analyzed using LC-HRMS composed of an 1290 Infinity II System (Agilent, Santa Clara, CA, USA) and X500R QTOF (Sciex, Framingham, MA, USA). The separation was achieved using a Zorbax Eclipse Plus C18 RRHD 1.8 µm, 50 mm × 2.1 mm, chromatographic column (Agilent). The column was kept at 40 °C. The eluents were water with 0.1% formic acid (Sigma Aldrich, Merck KGaA, Darmstadt, Germany) and methanol (Honeywell, Bartelt, Austria) with 0.1% formic acid. The flow of the eluent was 0.5 mL/minute. The gradient started at 50% of eluent B and was increased to 100% B within 3 min. Then, it was kept at 100% for 1 min, followed by 1 min equilibration at the initial concentration. This gradient effectively separates 3-hydroxymyristic acid (RT = 2.6 min) from 2-hydroxymyristic acid (RT = 2.7 min). The injection volume was 3 µL. The ionization was achieved using electrospray ionization in a negative mode at 500 °C and spray voltage at −4500 V. The declustering potential was set to −80 V. The mass spectrometer was operated in a scan mode from 150 to 350 Da. Data analysis was performed using Sciex OS Software (Version 1.7.0.36606). For the quantitation of 3-OH C14:0, the mass range of 243.19 ± 0.01 Da was extracted.

The endotoxin-derived esterified 3-OH C14:0 concentration was calculated by the difference of the total 3-OH C14:0 concentration (hydrolyzed sample) and the free unesterified 3-OH C14:0 concentration (sample without hydrolysis).

## 6. Statistics

Statistical analyses were performed with GraphPad Prism Version 9.3.1. (GraphPad Software, San Diego, CA USA). Pen was used as the experimental unit for the performance data, while animals was used as the experimental unit for all other parameters. The data were tested for normal distribution with the Shapiro–Wilk test. If the data were normally distributed, ANOVA was performed followed by Tukey’s multiple comparisons test. The Kruskal–Wallis test was used as a non-parametric test, if the data were not normally distributed. Differences were considered statistically significant when the *p*-value was < 0.05.

## Figures and Tables

**Table 1:** Body weight gain of broilers fed a control diet, a deoxynivalenol (DON)-contaminated diet, or a fumonisin (FB)-contaminated diet for 25 days. Animals were kept under thermoneutral conditions. *n* = 6 pens/treatment. Values are presented as mean with standard deviation.

Groups	Body Weight (g)	Body Weight Gain (g)		
	Day 1	Day 1–12	Day 12–24	Day 1–24
Control	43 ± 0	270 ± 8 ^a^	770 ± 21	1040 ± 29 ^a^
DON	43 ± 0	235 ± 8 ^b^	742 ± 12	977 ± 16 ^ab^
FBs	43 ± 1	243 ± 7 ^ab^	707 ± 22	950 ± 25 ^b^

^ab^ Superscripts indicate significant difference (*p* < 0.05).

**Table 2:** Gene expression data (CNRQ) of the mid-duodenum, mid-jejunum, and mid-ileum of broilers fed a control diet, a deoxynivalenol (DON)-contaminated diet, or a fumonisin (FB)-contaminated diet for 25 days. Animals were kept under thermoneutral conditions. *n* = 6 animals/treatment. Values are presented as mean with standard deviation.

	Gut Barrier Function														
	Mid-Duodenum					Mid-Jejunum					Mid-Ileum				
	CLDN1	CLDN5	OCLN	ZO1	ZO2	CLDN1	CLDN5	OCLN	ZO1	ZO2	CLDN1	CLDN5	OCLN	ZO1	ZO2
Control	0.72 ± 0.318	0.72 ± 0.142	0.83 ± 0.375	0.93 ± 0.1851	0.88± 0.279	0.76 ± 0.172	0.72 ± 0.177 ^a^	0.83 ± 0.523 ^a^	0.96 ± 0.814	0.89 ± 0.404 ^a^	0.86 ± 0.096	0.85 ± 0.042	1.03 ± 0.533	0.90 ± 0.158	0.83 ± 0.079 ^x^
DON	1.45 ± 0.906	0.93 ± 0.184	1.092 ± 0.299	1.068 ± 0.332	0.87 ± 0.172	0.60 ± 0.445	0.51 ± 0.254 ^ab^	0.51 ± 0.206 ^ab^	1.00 ± 0.581	0.30 ± 0.109 ^ab^	0.86 ± 0.431	0.83 ± 0.153	0.87 ± 0.451	0.95 ± 0.198	1.13 ± 0.265 ^y^
FBs	1.14 ± 0.622	0.99 ± 0.562	0.80± 0.260	0.74 ± 0.108	0.71 ± 0.308	0.47 ± 0.216	0.27 ± 0.166 ^b^	0.24 ± 0.167 ^b^	0.89 ± 1.065	0.22 ± 0.100 ^b^	0.99 ± 0.296	1.11 ± 0.378	1.32 ± 0.371	1.05 ± 0.887	1.17 ± 0.449 ^xy^
*p*-value	0.1946	0.2029	0.2377	0.0629	0.4439	0.3820	0.0101	0.0184	0.7996	0.0022	0.3346	0.5585	0.2427	0.8017	0.0803
	Inflammatory Response														
	Mid-Duodenum					Mid-Jejunum					Mid-Ileum				
	TLR2	IL6	IL8			TLR2	TLR4	IL1b	IL6	IL8	TLR2	TLR4	IL1b	IL6	IL8
Control	0.54 ± 0.214	0.30 ± 0.118	0.98 ± 1.079			0.60 ± 0.310	1.14 ± 0.269 ^a^	0.52 ± 0.334	0.49 ± 0.294	0.65 ± 0.534	0.75 ± 0.287	1.01 ± 0.252	0.768 ± 0.634	1.02 ± 0.609	0.57 ± 0.155
DON	1.84 ± 1.993	5.00 ± 7.366	0.59 ± 0.196			0.97 ± 0.666	0.39 ± 0.092 ^ab^	1.24 ± 1.541	1.45 ± 1.393	0.88 ± 0.911	1.04 ± 0.676	0.97 ± 0.220	0.88 ± 0.607	1.69 ± 2.364	1.30 ± 0.756
FBs	2.173 ± 2.761	4.18 ± 6.982	1.25 ± 1.356			0.40 ± 0.237	0.23 ± 0.128 ^b^	0.77 ± 1.047	0.88 ± 0.683	0.46 ± 0.320	0.83 ± 0.467	1.02 ± 0.362	0.39 ± 0.160	0.66 ± 0.412	0.83 ± 0.641
*p*-value	0.1664	0.0519	0.9690			0.1390	0.0002	0.4018	0.3493	0.8508	0.8850	0.9006	0.1743	0.7251	0.4786

^ab^ Superscripts indicate significant difference (*p* < 0.05). ^xy^ Superscripts indicate a trend (*p* < 0.10).

**Table 3:** Gene expression data (CNRQ) of the mid-duodenum, mid-jejunum, and mid-ileum of broilers fed a control diet, a deoxynivalenol (DON)-contaminated diet or a fumonisin (FB)-contaminated diet for 25 days. Animals were kept under heat stress conditions (34 °C) on day 26 for 10 h. *n* = 6 animals/treatment. Values are presented as mean with standard deviation.

	Gut Barrier Function														
	Mid-Duodenum					Mid-Jejunum					Mid-Ileum				
	CLDN1	CLDN5	OCLN	ZO1	ZO2	CLDN1	CLDN5	OCLN	ZO1	ZO2	CLDN1	CLDN5	OCLN	ZO1	ZO2
Control	0.69 ± 0.287 ^a^	0.748 ± 0.379	0.80 ± 0.208	1.30 ± 0.672	1.27 ± 0.736	1.43 ± 2.078	1.45 ± 1.205 ^a^	0.97 ± 0.399 ^x^	1.32 ± 0.625	1.28 ± 0.572 ^a^	0.70 ± 0.225	0.76 ± 0.286 ^x^	0.82 ± 0.396	0.90 ± 0.326	0.86 ± 0.205 ^x^
DON	0.88 ± 0.427 ^ab^	0.96 ± 0.656	0.90 ± 0.200	0.97 ± 0.260	1.19 ± 0.488	0.50 ± 0.113	0.55 ± 0.243 ^ab^	0.65 ± 0.321 ^xy^	1.84 ± 0.587	0.65 ± 0.395 ^ab^	0.78 ± 0.174	1.04 ± 0.271 ^xy^	1.14 ± 0.441	1.15 ± 0.376	1.02 ± 0286 ^xy^
FBs	1.44 ± 0.594 ^b^	1.21 ± 0.103	1.58 ± 0.780	1.10 ± 0.348	1.39 ± 0.272	0.46 ± 0.242	0.28 ± 0.090 ^b^	0.40 ± 0.216 ^y^	1.03 ± 0.905	0.51 ± 0.108 ^b^	1.08 ± 0.776	1.15 ± 0.391 ^y^	1.33 ± 0.741	1.07 ± 0.626	1.74 ± 1.035 ^y^
*p*-value	0.0295	0.1680	0.1630	0.6374	0.3405	0.6183	0.0039	0.0528	0.6580	0.0211	0.5929	0.0574	0.2931	0.6219	0.0509
	Inflammatory Response														
	Mid-Duodenum					Mid-Jejunum					Mid-Ileum				
	TLR2	IL6	IL8			TLR2	TLR4	IL1b	IL6	IL8	TLR2	TLR4	IL1b	IL6	IL8
Control	0.567 ± 0.338 ^a^	0.32 ± 0.148 ^a^	2.26 ± 2.371			1.51 ± 1.801 ^x^	0.95 ± 0.426 ^a^	1.43 ± 1.067 ^a^	3.29 ± 5.793	2.62 ± 2.018 ^a^	0.905 ± 1.173	0.69 ± 0.148 ^a^	0.67 ± 0.395	1.30 ± 1.916	1.22 ± 0.485
DON	0.812 ± 0.541 ^ab^	1.18 ± 1.723 ^ab^	3.52 ± 3.712			0.48 ± 0.189 ^xy^	0.67 ± 0.374 ^ab^	0.51 ± 0.302 ^ab^	0.57 ± 0.48	0.65 ± 0.265 ^b^	0.570 ± 0.580	1.04 ± 0.232 ^ab^	2.83 ± 3.92	2.01 ± 1.950	1.67 ± 0.862
FBs	1.68 ± 0.862 ^b^	3.65 ± 2.909 ^b^	1.18 ± 0.761			0.35 ± 0.228 ^y^	0.40 ± 0.099 ^b^	0.26 ± 0.180 ^b^	0.56 ± 0.676	0.89 ± 0.463 ^ab^	0.91 ± 1.173	1.84 ± 1.210 ^b^	0.52 ± 0.378	0.68 ± 0.384	1.96 ± 1.081
*p*-value	0.0174	0.0208	0.4570			0.0802	0.0074	0.0168	0.9823	0.0312	0.5487	0.0003	0.1790	0.8040	0.3022

^ab^ Superscripts indicate significant difference (*p* < 0.05). ^xy^ Superscripts indicate a trend (*p* < 0.10).

**Table 4:** Selected genes and references analyzed in the duodenum, jejunum, and ileum.

Selected Genes	NCBI Reference Sequence
Gut barrier function	
Claudin 1 (CLDN1)	NM_001013611
Claudin 5 (CLDN5)	NM_204201
Occludin (OCLN)	XM_025144248
Zonula occludens 1 (ZO1)	XM_015278981
Zonula occludens 2 (ZO2)	XM_025144669
Inflammation	
Toll-like receptor 2 (TLR2)	XM_0469144
Toll-like receptor 4 (TLR4)	NM_001030693
Interleukin 1-beta (IL1β)	XM_046931582
Interleukin 6 (IL6)	NM_204628
Interleukin 8 (IL8)	NM_205498
Housekeeping genes	
Beta actin (ACTB)	NM_205518
Glyceraldehyde-3-phosphate dehydrogenase (GAPDH)	NM_204305
Glucose 6-phosphate dehydrogenase (G6PD)	M11100
Ribosomal protein L7 (RPL7)	XM_046910609

## Data Availability

The data analyzed during the current study are available from the corresponding author on reasonable request.
